# Impact of Early-Life Antibiotic Exposure on Gut Microbiome and Vaccine Immunogenicity in Infants: A Narrative Review

**DOI:** 10.3390/jcm15114161

**Published:** 2026-05-28

**Authors:** Karolina Babik, Zuzanna Bomze, Magdalena Szuba, Beata Borek-Dzięcioł, Bożena Kociszewska-Najman

**Affiliations:** 1 Students’ Scientific Association “ProNeo”, Department of Neonatology and Rare Diseases, University Clinical Centre, Medical University of Warsaw, 02-091 Warsaw, Poland; s086700@student.wum.edu.pl (Z.B.);; 2 Department of Neonatology and Rare Diseases, University Clinical Centre, Medical University of Warsaw, 02-091 Warsaw, Poland

**Keywords:** infant, anti-bacterial agents, gastrointestinal microbiome, vaccination, dysbiosis

## Abstract

**Background:** Neonatologists face a significant clinical challenge in balancing the life-saving effects of broad-spectrum antibiotics with an infant’s ability to develop long-term vaccine-induced immunity. During the critical neonatal window, the gut microbiota acts as an essential endogenous adjuvant that promotes immune maturation through Toll-like receptor signaling and the production of microbial metabolites such as short-chain fatty acids. **Methods:** This narrative review was based on a comprehensive search of the PubMed and Google Scholar databases for studies published between 2014 and 2025. The search focused on the relationships between neonatal antibiotic exposure, gut microbiome development, and vaccine-induced immune responses in infants. **Results:** Early-life antibiotic exposure disrupts immune maturation by causing a marked reduction in commensal bacteria, particularly *Bifidobacterium* and *Bacteroides*. Clinical and epidemiological evidence indicates that this antibiotic-driven dysbiosis leads to significantly lower antibody titers following routine vaccinations, including PCV13, Hib, and DTaP, with measurable effects persisting up to 15 months of age. While antibiotics may paradoxically enhance oral rotavirus vaccine responses in resource-constrained settings by reducing environmental enteric dysfunction, an undisturbed native microbiota remains the optimal foundation for robust immunological memory. **Conclusions:** These findings highlight the necessity of improving antibiotic stewardship and exploring microbiota-restoring interventions, such as targeted probiotics, to optimize infant vaccination schedules and protect long-term immune health. Empirical antibiotic treatment should be promptly terminated once sepsis has been clinically excluded to preserve the gut–immune axis.

## 1. Introduction

Neonatologists face a significant clinical challenge: balancing the life-saving effects of broad-spectrum antibiotics with the preservation of long-term vaccine-induced immunity in infants.

The first weeks of infant life are a developmental period characterized by rapid colonization of the intestines by microorganisms, which actively influences the maturation of the immune system. These processes are closely related. The native microbiota works as an endogenous adjuvant that improves humoral and cellular defenses [1]. Empirical antibiotic therapy for suspected early-onset sepsis is certainly necessary; however, it is often associated with an imbalance of gut microbiome. Treatment might cause dysbiosis, due to destroying commensal bacteria, such as *Bifidobacterium* and *Bacteroides* [2]. Longitudinal cohorts monitoring infants show that early antibiotic exposure consistently reduces antibody titers subsequent to standard immunizations, specifically the 13-valent pneumococcal conjugate vaccine (PCV13), *Haemophilus influenzae type b* (Hib), and the diphtheria–tetanus–pertussis DTP vaccines [3,4].

This narrative review summarizes current evidence regarding the impact of neonatal antibiotic exposure on the development of the gut–immune axis and subsequent vaccine immunogenicity. Particular attention is given to mechanisms linking microbiota disruption with impaired humoral and cellular immune responses, including alterations in Toll-like receptor signaling, microbial metabolite production, dendritic cell activation, and T-cell maturation. The review also discusses current clinical and epidemiological evidence, potential microbiota-restoring interventions, and implications for antibiotic stewardship in neonatal care.

The aim of this narrative review is to analyze current literature to evaluate the impact of neonatal antibiotic exposure on the development of the gut–immune axis. Furthermore, this study aims to determine how antibiotic-induced dysbiosis influences the immunogenicity and long-term efficacy of routine childhood vaccinations, providing a clinical rationale for improved antibiotic stewardship in neonatal care.

## 2. Methods

This narrative review was conducted using a structured literature search strategy to identify and synthesize current evidence on the impact of neonatal antibiotic exposure on the development of the gut–immune axis and vaccine-induced immune responses in infants. A comprehensive search of the literature was performed in PubMed and Google Scholar. The search was conducted on 5 April 2026.

The search strategy combined Medical Subject Headings (MeSH) and free-text terms related to neonatal antibiotic exposure, gut microbiota development, and vaccine immunogenicity. Keywords included: “neonatal antibiotics,” “early-life antibiotic exposure,” “gut microbiome,” “gut–immune axis,” “dysbiosis,” “*Bifidobacterium*,” “vaccine response,” “vaccine immunogenicity,” “infant vaccination,” “PCV13,” “Hib,” “ DTP,” “rotavirus vaccine,” “Toll-like receptor,” and “short-chain fatty acids,” combined using Boolean operators (AND, OR). Studies published between 2014 and 2025 in English were considered. Greater emphasis was placed on studies from the last decade; however, earlier publications were included where they contributed to understanding biological mechanisms or epidemiological context.

The inclusion criteria comprised original clinical studies (cohort, case–control, cross-sectional, and longitudinal studies) evaluating associations between neonatal antibiotic exposure, microbiome development, and vaccine-induced immune responses in infants. Experimental animal studies investigating microbiota-dependent immune maturation and vaccine efficacy were also included because they contributed to understanding the underlying biological mechanisms. Selected systematic reviews and meta-analyses were incorporated to contextualize the findings. Exclusion criteria included studies involving only adult populations, studies not related to neonatal or early-life antibiotic exposure, case reports, conference abstracts, and studies of limited methodological quality.

**Figure 1:**
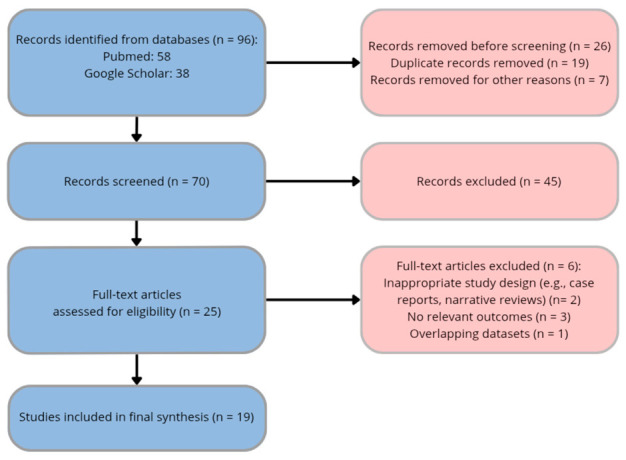
A flow diagram of the study selection process.

The study selection process was conducted in two stages by two independent reviewers. After removing duplicates, titles and abstracts were screened for relevance, followed by full-text assessment according to predefined eligibility criteria. Discrepancies were resolved by consensus. A total of 96 records were initially identified (PubMed: 58; Google Scholar: 38). After removing 19 duplicates and 7 records excluded for other reasons, 70 titles and abstracts were screened. Of these, 45 records were excluded as not directly relevant to the topic. The full texts of 25 articles were assessed for eligibility, of which 6 were excluded (inappropriate study design, n = 2; lack of relevant outcomes, n = 3; overlapping datasets, n = 1). Ultimately, 19 studies were included in the qualitative synthesis. The study selection process is summarized in Figure 1.

Due to heterogeneity in study design, antibiotic exposure definitions, microbiome assessment methods, and vaccine outcome reporting, findings were synthesized qualitatively rather than quantitatively. Data extraction was descriptive and focused on study design, population characteristics, exposure assessment, microbiome alterations, and vaccine-related immune outcomes.

The methodological quality of included studies was assessed narratively. Although no formal risk-of-bias tool was applied, key aspects such as study design, control of confounding factors, exposure measurement, microbiome analysis methods, and outcome assessment reliability were critically evaluated. This approach is consistent with the narrative nature of the review and represents a limitation that should be considered when interpreting the findings. No new human or animal studies were conducted; therefore, ethical approval was not required.

## 3. Antibiotic Exposure in the Neonatal Period—Epidemiology and Mechanisms of Microbiome Disruption

### 3.1. Extent of Antibiotic Use in Neonates

The extent of antibiotic use in neonates depends on the studied population. In cohort studies, including term neonates and late preterm infants, antibiotics were given to 1–3% of neonates during the first week of life [5,6]. In contrast, antibiotic use is much higher in Neonatal Intensive Care Units (NICUs), where antibiotics are the most frequently prescribed type of medications [7]. In a prospective cross-sectional study, Hematian et al. [8] showed that more than 84% of neonates treated in NICUs received antibiotics.

### 3.2. Reasons for Antibiotic Use in Neonates

Perinatal infections are one of the most common reasons for antibiotic use in neonates, especially during the first days of life. The main indication for starting antibiotic therapy in newborns is suspected early-onset sepsis (EOS) [9]. EOS is a serious bacterial infection that occurs within 72 h after birth and is confirmed by a positive blood or cerebrospinal fluid culture. The most common bacteria causing EOS are group B streptococci (GBS) and *Escherichia coli* [10,11]. Clinical guidelines recommend empirical treatment of all suspected EOS cases with a beta-lactam antibiotic (usually benzylpenicillin or ampicillin) combined with an aminoglycoside, most often gentamicin [12]. Studies have shown that the incidence of EOS ranges from 0.13 to 1.45 per 1000 live births; however, up to 58 times more neonates receive antibiotics due to the suspected disease [6]. The epidemiological landscape and clinical indications for antibiotic therapy in diverse neonatal populations are summarized in Table 1.

Late-onset sepsis (LOS) is sepsis that occurs after 72 h of life. LOS differs from EOS in pathogen type and source of infection. It is often acquired after birth, for instance in the hospital environment, and is associated with the use of catheters, parenteral nutrition, as well as contact with healthcare staff or parents [13]. The clinical presentation of LOS is variable and non-specific. Common symptoms include respiratory signs, lethargy, tachycardia, feeding intolerance, and temperature instability, such as fever or hypothermia. There are still no diagnostic tests that allow early diagnosis; therefore, antibiotics are often given empirically. The choice of antibiotics is based on local epidemiology and likely pathogens [14]. A large microbiological analysis showed that the main causes of LOS are *coagulase-negative Staphylococci* (CoNS). *Staphylococcus aureus* and *Enterococcus faecalis* were also isolated, while Gram-negative bacteria accounted for a smaller proportion of cases [15]. The most commonly used antibiotics in LOS include: vancomycin, mainly for suspected Gram-positive infections; gentamicin used in combination with other drugs; cefotaxime for coverage of Gram-negative pathogens; and meropenem, which is used in severe or resistant infections [16].

### 3.3. Most Commonly Used Antibiotic Classes in Neonates and Their Impact on Gut Microbiota Diversity

Various classes of antibiotics are used in the treatment and prevention of infections in neonates. Among the most commonly used, especially as empirical therapy for suspected neonatal sepsis, are beta-lactams such as ampicillin, beta-lactam/beta-lactamase inhibitor combinations (e.g., ampicillin/sulbactam), and cephalosporins (e.g., cefotaxime) [17]. Aminoglycosides, such as gentamicin and amikacin, are often used together with beta-lactams. They increase treatment effectiveness due to their activity against Gram-negative pathogens. In severe or resistant infections, meropenem is often used [18].

Studies show that antibiotic use during the neonatal period is associated with reduced gut microbiota diversity, which may affect immune system development and metabolism [19]. Antibiotics given to newborns do not act only on pathogens, but also disrupt beneficial microorganisms, including *Bifidobacterium* species [20]. These Gram-positive bacteria naturally colonize the neonatal gut, especially in breastfed infants. They are essential for proper development and support lactose metabolism. *Bifidobacterium* also helps prevent colonization by opportunistic pathogens, such as Klebsiella and Enterococcus. A cohort study showed that antibiotic therapy during the first weeks of life reduced the abundance and diversity of Bifidobacteria up to the age of two years. Antibiotic treatment may also disrupt metabolic functions of the microbiome: production of metabolites, such as short-chain fatty acids, which are important for gut development and immune barrier function. In the mentioned study, Xu et al. [21] demonstrated that early, short-term antibiotic exposure significantly disrupted the proper development of neonatal microbiome and its functional capacity. In infants who did not receive antibiotics, the microbiota became more diverse and functionally rich during the first three weeks of life. In contrast, antibiotic exposure changed this trajectory, and the microbiome did not follow the typical pattern of development, which suggests delayed or impaired maturation of metabolic functions.

### 3.4. Consequences of Gut Microbiome Dysbiosis for Immune System Maturation

Gut microbiota dysbiosis after early-life antibiotic therapy leads to long-term changes in bacterial composition, which disrupt normal immune system maturation. Dysbiosis is associated with increased numbers of CD4^+^ T cells, CD8^+^ T cells, and Th17 cells, together with a reduced number of regulatory T cells (CD4^+^CD25^+^Foxp3^+^ Treg). As a result, the balance between pro-inflammatory immune responses and immune tolerance is impaired. Disturbed immune maturation in infants and young children may increase susceptibility to infections due to poor regulation of immune responses. It is also associated with the risk of immune-related diseases later in life, such as inflammatory bowel disease, allergic diseases, asthma, and metabolic disorders linked to chronic inflammation [22].

## 4. Development of the Gut–Immune Axis in the Neonatal Period

### 4.1. Early Microbial Colonization and Immune Maturation

The neonatal period represents a critical window of immune system development during which the intestinal microbiota plays a key role in shaping immune maturation and responses to vaccines in the first year of life [23]. At birth, infants are sterile and rapidly colonized by maternal and environmental microbes. This process begins immediately after delivery and is influenced by factors such as delivery mode, feeding practices, gestational age, and early antibiotic exposure. The initial microbiota provides essential signals for proper immune maturation [23,24,25].

Early interactions between gut microbiota and the host immune system involve microbial components, epithelial cells, and immune cells within gut-associated lymphoid tissue. Intestinal epithelial cells (IEC) maintain gut homeostasis and produce cytokines such as IL-25 and thymic stromal lymphopoietin, as well as retinoic acid and transforming growth factor-β, which regulate immune responses. Through these mechanisms, IEC indirectly influence vaccine responses by shaping antigen presentation and immune maturation during the neonatal period [23,25].

### 4.2. Toll-like Receptor Signaling and Immune Education

Commensal microorganisms interact with the neonatal immune system primarily through pattern recognition receptors, including Toll-like receptors (TLRs), expressed on intestinal epithelial cells and antigen-presenting cells. These interactions are mediated by microbial-associated molecular patterns (MAMPs), which provide essential signals for immune education during early life [23,25,26,27].

TLR signaling induced by MAMPs promotes dendritic cell maturation and regulates cytokine production, supporting the development of antigen-specific immune responses [26,27]. In neonates, balanced TLR activation is particularly important, as excessive or insufficient signaling may impair immune education, resulting in suboptimal responses to vaccine antigens administered during infancy [28].

### 4.3. Role of Microbial Metabolites: Short-Chain Fatty Acids (SCFAs)

Gut microbiota-derived metabolites, especially SCFAs such as acetate, propionate, and butyrate, play a key role in immune regulation during early life [29]. SCFAs have been shown to significantly influence the development and differentiation of T cells, including regulatory (Treg) and helper (Th) subsets, as well as the maturation of B cells responsible for IgA and IgG antibody production [28]. Additionally, SCFAs modulate dendritic cell function and cytokine secretion, affecting antigen processing and presentation after vaccine administration [29].

### 4.4. Dendritic Cells as Mediators Between Microbiota and Vaccine Responses

Dendritic cells serve as a critical link between gut microbiota-derived signals and adaptive immune activation in early life [26]. Through TLRs and other pattern recognition receptors, dendritic cells sense MAMPs derived from commensal bacteria and integrate these signals with vaccine antigens [23,26]. Microbiota-derived ligands such as lipopolysaccharide and flagellin, act as endogenous adjuvants by activating TLR4 and TLR5 pathways. This activation promotes dendritic cell migration to draining lymph nodes and supports efficient T-cell priming. Proper dendritic cell function is required for T follicular helper cell differentiation and the generation of long-lasting antibody responses after infant vaccination [30].

### 4.5. Secretory Immunoglobulin A (Siga) and Microbiota

Secretory immunoglobulin A is the main antibody at mucosal surfaces, preventing microbial adhesion and neutralizing antigens without causing inflammation [23,31]. IgA production is thought to be influenced by the sampling of luminal commensal bacteria or their antigens by M cells, which are interspersed within the epithelial layer of the mucosa, and subsequently processed by dendritic cells in Peyer’s patches [23]. Neonatal Siga, including maternal IgA from breast milk, shapes early microbiota by limiting pathogenic taxa and supporting beneficial commensals [32].

### 4.6. Regulatory T Cells (Treg) and Microbiota

Foxp3+ regulatory T cells (Tregs) control immune tolerance in the neonatal gut, limiting inflammation during early microbial colonization [23,32]. Commensal bacteria and metabolites such as SCFAs promote Treg differentiation and enhance their suppressive function [23].

**Figure 2:**
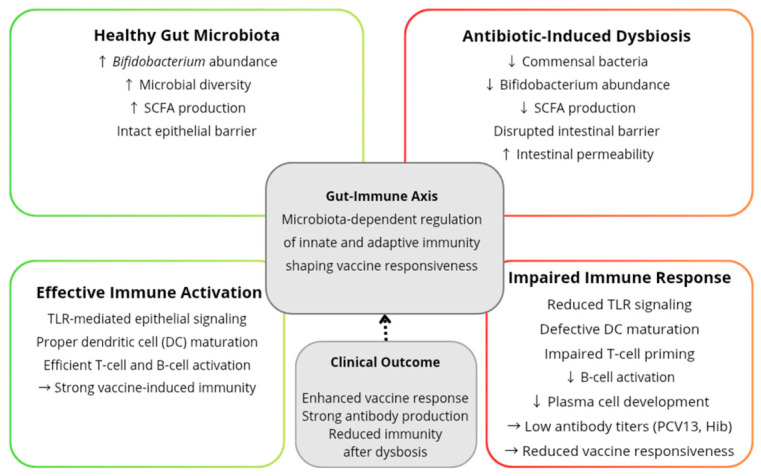
Mechanistic model of the gut–immune axis and the impact of antibiotic-induced dysbiosis on vaccine responsiveness. The left panel illustrates a healthy gut microbiota characterized by high microbial diversity, abundance of *Bifidobacterium*, and increased production of short-chain fatty acids (SCFAs), promoting proper toll-like receptor (TLR)-mediated signaling, dendritic cell (DC) maturation, and efficient adaptive immune activation, ultimately resulting in robust vaccine-specific antibody responses. The right panel depicts antibiotic-induced dysbiosis associated with loss of commensal bacteria, reduced SCFA production, impaired epithelial barrier integrity, defective immune signaling, and diminished antibody responses to vaccines such as pneumococcal conjugate vaccine (PCV13) and *Haemophilus influenzae type b* (Hib). Abbreviations: SCFAs, short-chain fatty acids; TLR, toll-like receptor; DC, dendritic cell; PCV13, 13-valent pneumococcal conjugate vaccine; Hib, *Haemophilus influenzae type b*. The arrows ↑, ↓ and → indicate an increase, a decrease, and a causal transition/direction, respectively.

Breastfeeding supports Treg expansion, while early-life dysbiosis or antibiotic exposure reduces Treg numbers, impairing immune regulation [33]. Proper Treg function is essential for balanced immune responses to vaccines, as it regulates germinal center formation and antibody quality [32]. The complex interplay between the gut microbiota and the developing immune system, and the detrimental effects of antibiotic-induced dysbiosis on vaccine responsiveness, are conceptually summarized in Figure 2.

### 4.7. Experimental Evidence from Animal Models

Experimental studies provide strong evidence that early-life disruption of gut microbiota impairs vaccine efficacy. Lynn et al. [2] demonstrated that antibiotic-driven intestinal dysbiosis in neonatal mice resulted in significantly impaired antibody responses to multiple vaccines compared with mice unexposed to antibiotics. These effects were associated with altered cytokine production, impaired dendritic cell signaling, and disrupted T-cell-dependent immune responses. Notably, similar antibiotic exposure in adult mice did not result in immune defects, emphasizing the unique vulnerability of the neonatal immune system.

**Figure 3:**
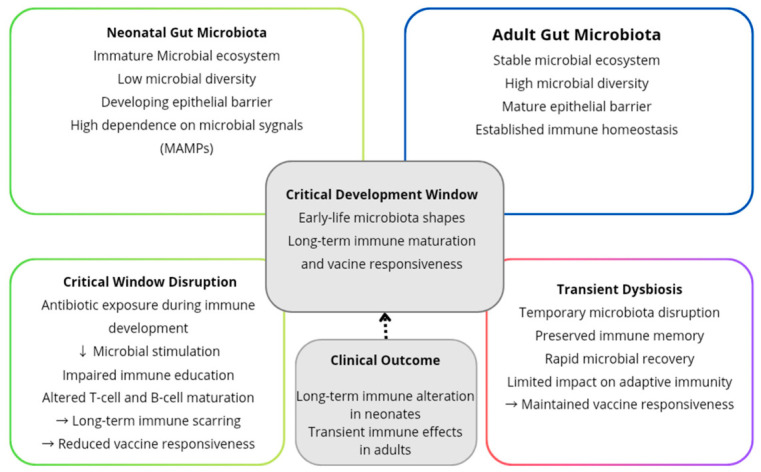
Differential impact of antibiotic exposure during neonatal and adult immune development. The schematic compares the consequences of antibiotic exposure during the neonatal critical developmental window versus adulthood. In neonates, disruption of the immature gut microbiota leads to reduced microbial-associated molecular pattern (MAMP) signaling, impaired immune education, and long-term immune dysregulation (“immune scarring”), resulting in reduced vaccine responsiveness. In contrast, adults possess a stable and mature microbiota with established immune homeostasis, where antibiotic-induced dysbiosis is typically transient and has limited long-term effects on adaptive immune memory and vaccine responses. Abbreviations: MAMPs, microbial-associated molecular patterns. The arrows ↓ and → indicate an decrease, and a causal transition/direction, respectively.

The unique vulnerability of the neonatal immune system extends beyond microbiome immaturity alone. Neonates physiologically exhibit reduced antigen-presenting cell function, diminished type I interferon responses, impaired T-helper cell polarization, and limited germinal center formation compared with adults [34,35]. In early life, immune responses are additionally biased toward tolerance in order to avoid excessive inflammation during initial microbial colonization. Consequently, disruption of microbiota-derived immune signaling during this developmental window may have disproportionately greater effects on vaccine-induced immune maturation than similar perturbations occurring in adulthood [23,32]. The distinct impact of antibiotic exposure during the critical neonatal developmental window compared to the more resilient adult immune system is visually represented in Figure 3.

## 5. Evidence from Clinical and Epidemiological Studies

### 5.1. Vaccine Immunity

Giving infants antibiotics in their early life affects their gut microbiome and significantly reduces the effectiveness of routine childhood vaccinations [3,34]. When neonates receive antibiotic treatments, they subsequently fail to produce adequate antibody titers against critical pathogens, such as *Streptococcus pneumoniae*, *Haemophilus influenzae type b*, and diphtheria/tetanus toxoids [3]. This antibody deficiency is not transient and remains measurable even at 7 and 15 months of age [3]. Mouse models exploring this dynamic reveal that a strong PCV13 response fundamentally requires an intact microbiota, a condition that researchers could actually restore by intentionally supplying the animals with *Bifidobacterium* strains [3]. Longitudinal observation confirms this trend for children who receive antibiotics during their first two years of life [36]. With every additional course of antimicrobial therapy, pediatric patients experienced statistically significant drops in their antibody levels, both before and after receiving diphtheria, tetanus and acellular pertussis (DTaP), Hib, inactivated polio vaccine (IPV), and PCV boosters [36].

### 5.2. Oral Vaccines

Orally administered vaccines depend on direct mucosal engagement within the gastrointestinal system, which means that any modifications to the resident microbiota can substantially compromise their protective efficacy [37]. The Etiology, Risk Factors and Interactions of Enteric Infections and Malnutrition and the Consequences for Child Health and Development (MAL-ED) cohort study shows this relationship when assessing rotavirus immunizations across low- and middle-income demographics [37]. St. Jean and colleagues reported a seemingly contradictory outcome: neonatal antibiotic exposure correlated with a 40% increase in the likelihood of vaccine seropositivity, standing in contrast to the immunosuppressive effects observed with intramuscular injections [37]. This observation is most likely explained by the baseline health status of the newborns in resource-constrained settings, who often experience environmental enteric dysfunction alongside a high burden of competing intestinal pathogens [37]. In this specific clinical conditions, broad-spectrum antibiotics eliminate the pathological bacterial overgrowth and reduce mucosal inflammation; this creates a temporary physiological window during which the live-attenuated viral strain can successfully replicate and provoke a measurable serologic response [37]. However, microbiome evaluations in healthy pediatric population confirm that keeping of the native gut flora remains the optimal biological condition [25]. Infants with high levels of natural gut commensals (especially *Bifidobacterium* and *Bacteroides* species) generate superior baseline humoral and cellular immunity after following standard immunization schedules [25]. In general, the intestinal architecture serves as an independent prognostic factor for future vaccine efficacy, regardless of whether that environment has been modified by early pharmacological interventions or underlying pathogenic colonization [25].

This paradoxical enhancement appears to be particularly relevant for live-attenuated oral vaccines, such as rotavirus vaccines, which require limited replication within the intestinal mucosa to induce protective immunity [38]. By temporarily reducing competing enteropathogens and intestinal inflammation, antibiotics may facilitate replication of the vaccine strain under specific pathological conditions. In contrast, a similar effect would be less likely for inactivated oral vaccines, which do not depend on active intestinal replication and are generally more dependent on intact mucosal antigen presentation pathways [25,38].

A detailed comparison of the quantitative impact of antibiotics on the immunogenicity of various vaccine types is presented in Table 2.

### 5.3. Cellular Mechanisms

Researchers mapping the presence of *Bifidobacterium* during early infancy discovered a direct correlation between higher bacterial counts and much stronger immunological memory against hepatitis B, tetanus toxoid, and tuberculosis [36]. Timing is very important here, since the proliferation of *Bifidobacterium* during the primary vaccination window (usually between 6 and 15 weeks of age) initiates a critical immune cascade [36]. At two years old, they not only have high amounts of antibodies, but their T-cells are also proliferating very quickly [36]. When infants receive antibiotics, the drugs disrupt this important colonization process and actively stop the development of the immune system [3,34,36]. The removal of microorganisms impairs the strong, long-lasting biological response necessary for vaccines to be effective [3,25,34,36].

Importantly, the immunomodulatory effects of probiotics appear to be highly strain-specific. Experimental and translational studies most frequently support the role of *Bifidobacterium longum* subsp. *infantis* and *Bifidobacterium* breve, which demonstrate enhanced intestinal colonization capacity, increased short-chain fatty acid production, and beneficial effects on immune maturation during early life [40]. These observations suggest that targeted microbiota-restoring interventions may represent a promising strategy for preserving vaccine responsiveness in antibiotic-exposed infants.

### 5.4. Other Commensal Microorganisms and Microbiome Diversity

Although *Bifidobacterium* and *Bacteroides* are among the most extensively studied taxa associated with vaccine responsiveness, increasing evidence suggests that broader microbiome composition and microbial diversity also influence immune maturation and vaccine efficacy [23,25,29]. Huda et al. [1] demonstrated that increased abundance of *Clostridiales*, *Enterobacteriales*, and *Pseudomonadales* in early infancy was associated with systemic inflammation and lower responses to oral and parenteral vaccines, whereas predominance of *Actinobacteria*, particularly *Bifidobacterium* species, correlated with stronger vaccine-induced immunity and larger thymic size.

Additional prospective evidence indicates that early-life microbiome composition may influence antibody responses to pneumococcal and tetanus vaccines during infancy. Moroishi et al. [25] identified significant associations between beta diversity of the infant gut microbiome and vaccine-specific IgG responses at 12 months of age. Metagenomic analyses further suggested that microbial metabolic pathways, rather than taxonomic composition alone, may contribute to variability in vaccine responsiveness.

Importantly, microbial diversity in early life has also been linked to broader immune maturation processes. Jakobsson et al. [41] showed that reduced gut microbiota diversity and delayed colonization by *Bacteroidetes* in infants delivered by Caesarean section were associated with impaired Th1-associated immune responses during the first two years of life. Collectively, these findings suggest that both microbiome composition and functional microbial diversity may play important roles in shaping vaccine-induced immune responses during infancy.

### 5.5. The Gut Virome and Mycobiome

Beyond the bacterial community, the neonatal gut is a complex ecosystem comprising viruses and fungi, which are also susceptible to antibiotic-induced perturbations [42,43]. The assembly of the gut virome begins shortly after birth and is initially dominated by bacteriophages that exhibit high dynamic variability during the first weeks of life [44]. Longitudinal metagenomic analyses of preterm infants have shown that neonatal antibiotic therapy (e.g., ampicillin and gentamicin) significantly alters the successional patterns of the virome, leading to a decrease in viral diversity and shifts in phage–host interactions [42]. These changes in the virome landscape may independently modulate the host immune system through interactions with pattern recognition receptors [44]. Similarly, the gut mycobiome undergoes rapid development during the first month of life, with its composition being shaped by delivery mode and clinical interventions [43]. Research indicates that *Candida* and *Saccharomyces* are among the primary fungal colonizers of the neonatal gut [43]. Antibiotic exposure can disrupt the balance between bacteria and fungi, potentially leading to fungal overgrowth or dysbiosis, which has been linked to altered immune maturation pathways [42]. Although the direct impact of these non-bacterial shifts on specific vaccine antibody titers remains an area for future investigation, the disruption of the multi-kingdom microbial network underscores the broader ecological cost of neonatal antibiotic use [42,44].

## 6. Conclusions and Future Directions

The primary objective of this review was to evaluate the impact of neonatal antibiotic exposure on the development of the gut–immune axis and subsequent vaccine immunogenicity. While broad-spectrum antibiotics are still important for treating neonatal sepsis, their ecological cost is undeniable. Depleting key commensal bacteria, like *Bifidobacterium*, impairs the gut–immune axis, fracturing TLR signaling and stunting T-cell proliferation. As a result, this early dysbiosis prevents children from being able to build persistent antibody responses to common intramuscular vaccines, including PCV13, Hib, and DTaP. To address these findings and fulfill the study’s aim of optimizing infant health, it is imperative to exercise greater clinical diligence in adhering to diagnostic standards for initiating therapy. Crucially, empirical antibiotic treatment should be promptly terminated once early-onset sepsis (EOS) has been clinically excluded and blood or cerebrospinal fluid cultures remain negative. Although antibiotics may paradoxically enhance oral rotavirus vaccine responses in resource-constrained settings, maintaining an undisturbed native microbiota remains the optimal foundation for robust immunological memory. Therefore, improving antibiotic stewardship and exploring microbiota-restoring interventions, such as targeted *Bifidobacterium* supplementation, are vital strategies for preserving vaccination efficacy in vulnerable newborns. Importantly, probiotic activity varies between strains, and different *Bifidobacterium* species may have distinct effects on immune system development. Clinical investigations evaluating specialized *Bifidobacterium* supplementation, in conjunction with necessary antibiotic regimens, present a viable approach to reconstruct intestinal architecture, thereby preserving vaccination immunogenicity for extremely susceptible newborns.

### Clinical Perspective and Take-Home Message

A critical priority for neonatologists involves reconciling the essential use of life-saving antibiotics with the need to safeguard an infant’s future immune response to vaccinations. While empirical therapy for suspected early-onset sepsis is necessary, it is often applied far more frequently than infections are confirmed, with up to 58 times more neonates receiving treatment than those with positive cultures. Current evidence indicates that antibiotic-induced dysbiosis, specifically the depletion of commensal bacteria such as *Bifidobacterium* and *Bacteroides*, disrupts the gut–immune axis and leads to significantly lower antibody titers for routine vaccinations, including PCV13, Hib, and DTaP. Although antibiotics may paradoxically enhance oral rotavirus vaccine responses in specific resource-constrained settings by reducing environmental enteric dysfunction, an undisturbed native microbiota remains the optimal foundation for robust immunological memory.

The integrity of the neonatal gut microbiome is a fundamental determinant of the strength and longevity of vaccine-induced immune responses. In clinical practice, priority should be given to rigorous antibiotic stewardship, including the prompt termination of empirical treatment once sepsis is clinically excluded and cultures remain negative. Future strategies involving targeted *Bifidobacterium* supplementation represent a promising approach to reconstruct intestinal architecture and preserve vaccination efficacy in vulnerable newborns requiring antimicrobial therapy. Current evidence remains insufficient to define standardized supplementation protocols, and further randomized clinical trials are required to determine optimal strains, timing, and duration of administration.

## Figures and Tables

**Table 1:** Antibiotic utilization rates and clinical indications across neonatal populations.

Patient Group/Indicator	Prevalence/Ratio	Primary Indication
Term and Late Preterm Infants	1–3% of all neonates	Primarily administered during the first week of life due to suspected early-onset sepsis (EOS).
Neonatal Intensive Care Units (NICU)	>84% of hospitalized neonates	Antibiotics are the most frequently prescribed type of medications in NICUs, used empirically for suspected EOS and LOS.
Treatment vs. Confirmation Ratio	58:1	Up to 58 times more neonates receive antibiotics for suspected EOS than those with a confirmed positive culture.

NICU—Neonatal Intensive Care Unit; EOS—Early-Onset Sepsis; LOS—Late-Onset Sepsis.

**Table 2:** Quantitative impact of early-life antibiotic exposure on vaccine-induced immune responses.

Author and Year	Vaccine Type	Key Quantitative Finding/Timeline
**Ryan et al. (2025)** [3]	PCV13, Hib, DTaP	Significantly lower IgG GMCs against 6 PCV13 serotypes and Hib-PRP at 7 months. By 15 months, seroprotection rates were lower for all 13 PCV13 serotypes in infants exposed to antibiotics compared to the no-antibiotic group.
**St. Jean et al. (2022)** [37]	Oral Rotavirus	A 40% increase in the likelihood of vaccine seropositivity (PR: 1.40, 95% CI: 1.04–1.89). A hypothetical intervention removing inappropriate antibiotics would result in a 4% absolute reduction in seropositivity.
**Huda et al. (2019)** [39]	HBV, Tetanus, BCG	*Bifidobacterium* abundance during the 6–15 week window correlates with TT-specific CD4 T-cell proliferation (R = 0.33, P = 0.034) and cellular immunity at 2 years of age.
**Chapman et al. (2022)** [36]	DTaP, Hib, IPV, PCV	Every additional antibiotic course reduces pre-booster antibody levels by 5.8–11.3% and post-booster levels by 18.1–21.3% (all *p* < 0.05).

BCG—Bacillus Calmette-Guérin; DTaP—Diphtheria, tetanus and acellular pertussis; GMCs—Geometric Mean Concentrations; HBV—Hepatitis B virus; Hib—*Haemophilus influenzae type b*; IgG—Immunoglobulin G IPV—Inactivated polio vaccine; PCV/PCV13—13-valent pneumococcal conjugate vaccine; PRP—Polyribosylribitol phosphate; TT—Tetanus toxoid.

## Data Availability

Data sharing is not applicable to this article as no new datasets were created or analyzed during the current study.
